# Feasibility and usefulness of video-based markerless two-dimensional automated gait analysis, in providing objective quantification of gait and complementing the evaluation of gait in children with cerebral palsy

**DOI:** 10.1186/s12891-024-07853-9

**Published:** 2024-09-17

**Authors:** Evelina Pantzar-Castilla, Diletta Balta, Ugo Della Croce, Andrea Cereatti, Jacques Riad

**Affiliations:** 1 Department of Orthopedics, Orebro University School of Medical Sciences and Orebro University Hospital, Orebro, Sweden; 2 Department of Electronics and Telecommunications, Politecnico di Torino, Corso Castelfidardo, 39, Torino, 10129 Italy; 3 Department of Biomedical Sciences, University of Sassari, Viale San Pietro 43b, Sassari, Italy; 4 Department of Orthopedics, Skaraborg Hospital Skövde, Lovangsvagen 541 42, Skövde, Sweden; 5 Institute of Clinical Sciences, Sahlgrenska Academy, University of Gothenburg, 405 30, Gothenburg, Sweden

**Keywords:** Cerebral palsy, Gait, 2-dimensional markerless gait analysis, Follow-up program

## Abstract

**Background:**

Gait analysis aids in evaluation, classification, and follow-up of gait pattern over time in children with cerebral palsy (CP). The analysis of sagittal plane joint kinematics is of special interest to assess flexed knee gait and ankle joint deviations that commonly progress with age and indicate deterioration of gait. Although most children with CP are ambulatory, no objective quantification of gait is currently included in any of the known international follow-up programs. Is video-based 2-dimensional markerless (2D ML) gait analysis with automated processing a feasible and useful tool to quantify deviations, evaluate and classify gait, in children with CP?

**Methods:**

Twenty children with bilateral CP with Gross Motor Function Classification Scale (GMFCS) levels I–III, from five regions in Sweden, were included from the national CP registry. A single RGB-Depth video camera, sensitive to depth and contrast, was positioned laterally to a green walkway and background, with four light sources. A previously validated markerless method was employed to estimate sagittal plane hip, knee, ankle kinematics, foot orientation and spatio-temporal parameters including gait speed and step length.

**Results:**

Mean age was 10.4 (range 6.8–16.1) years. Eight children were classified as GMFCS level I, eight as II and four as III. Setup of the measurement system took 15 min, acquisition 5–15 min and processing 50 min per child. Using the 2D ML method kinematic deviations from normal could be determined and used to implement the classification of gait pattern, proposed by Rodda et al. 2001.

**Conclusion:**

2D ML assessment is feasible, since it is accessible, easy to perform and well tolerated by the children. The 2D ML adds consistency and quantifies objectively important gait variables. It is both relevant and reasonable to include 2D ML gait assessment in the evaluation of children with CP.

## Introduction

### Flexed knee gait and the consequences of progression

In ambulatory children with cerebral palsy (CP), flexed knee gait is a frequently described problem [1–4]. With growth and increased weight, muscles become short and unbalanced, hip, knee and ankle joint deformities develop and rotational malalignments, including foot deformities, become evident, influencing gait negatively [2, 5]. There are several reports on the deterioration of gait and physical function in adolescence and adulthood in CP [3, 4, 6]. One of the challenges in care of ambulatory children with CP, from an orthopedic point of view, is early identification of gait deviations and progression, enabling intervention with adequate treatment [1, 7–9].

### Follow-up program

In Sweden, which has a population of 10 million inhabitants, the cerebral palsy follow-up program (CPUP) encompasses more than 95% of children with CP [10, 11]. Children are followed regularly by a local rehabilitation team and are examined at specific intervals depending on age and Gross Motor Function Classification Scale (GMFCS) level [12, 13]. A physiotherapist and occupational therapist perform physical examinations of passive joint range of motion and assess spasticity and function of the lower and upper extremity [14]. The orthopedic surgeon is responsible for obtaining radiographs of the hips and spine in accordance with a standardized protocol [14]. The pediatric neurologist provides overall medical care and has an important role as a coordinator in the rehabilitation organization. The objective of CPUP is to prevent hip dislocation and severe deformities and provide treatment at an early stage, thereby optimizing the functional ability and quality of life in children with CP [12]. Registers with similar programs has been introduced in several countries [15–17].

Although most children with CP are ambulatory, no objective quantification of gait is currently included in any of the known international follow-up programs.

### Gait analysis

Gait analysis is important to identify, quantify and follow gait deviations over time and to evaluate treatment [1, 3, 6, 18–20]. Three-dimensional gait analysis (3D GA) is considered the gold standard, giving a detailed description of gait, although not frequently available due to the cost and need for a dedicated space [21–23]. Furthermore, for follow-up and screening purposes, the high level of details provided by 3D GA might be unnecessary and overly complex. Moreover, the analysis of sagittal plane kinematics may be sufficient [24–26]. Less advanced (and less costly) systems have been developed, such as the Edinburgh Visual Gait Score, specifically developed for assessing gait in CP. However, the Edinburgh Visual Gait Score requires manual processing, making it time-consuming [24, 27–30]. In recent years, two-dimensional markerless (2D ML) gait analysis methods have been developed and validated in both healthy individuals as well as in children with CP for the sagittal plane kinematics [31–36]. With advances in video and depth technology (RGBD) and by incorporating automated processing algorithms, this methodology has become less time-consuming and does not require expensive hardware. When using a single pre-calibrated RGBD camera, the 2D ML system becomes easy to handle, set up and can be used in children’s regular assessment locations.

Previous research, while extensive, focuses on treatment, mainly surgical, of flexed knee gait and knee flexion contractures in CP [2–4]. One of the main difficulties in the management of children with CP is early identification and detection of those at risk of developing deterioration of gait, including flexed knee gait pattern.

Rodda et al. have presented a classification of gait patterns and treatment algorithms for ambulatory children, including the changes commonly described with increasing age [3, 4]. The classification in bilateral and unilateral CP is based mainly on sagittal plane kinematics derived from a 3D GA system [3]. However, the absence of quantitative information on sagittal plane kinematics in everyday practice limits the use of classification systems in follow-up programs over time. In Sweden, there is a well-established follow-up program that encompasses most children with CP. It would be feasible and beneficial for the ambulatory children to have supplementary, quantitative evaluation of gait.

### Gait pattern classification by Rodda et al

The classification system of Rodda et al. is based on kinematics in the sagittal plane for children with bilateral and unilateral CP, using primarily the position of the foot, secondarily the kinematics of the ankle and knee, and lastly the kinematics of the hip and pelvis, to cover the full complexity of spastic gait disorders [3]. The classification is organized so it follows the pattern common with increased age as well as including treatment options [3].

#### Type I

True equinus gait pattern, toe-walking with ankle plantarflexion throughout stance is the dominant sign with the knee and hip in extension.

#### Type II

Jump gait pattern, toe-walking with the ankle in plantarflexion and the knee and hip in flexion.

#### Type III

Apparent equinus gait pattern, toe-walking, with normal range of ankle dorsiflexion and increased knee and hip flexion.

#### Type IV

Crouch gait pattern, excessive ankle dorsiflexion and excessive knee and hip flexion [3].

### Aim

The aims of this study were to explore, the feasibility of using the 2D ML system at local rehabilitation clinics in Sweden, and discuss the usefulness in the evaluation and classification of gait, in children with CP.

## Methods

### Participants and procedure

Approval was obtained from the regional ethical review board in Gothenburg, Sweden (1134-16). Children aged 4–18 years, living in any of five regions in Sweden (Skaraborg, Närke, Västmanland, Dalarna and Hälsingland), were identified from the CPUP registry. Those eligible for inclusion were sent a letter with information and invitation to participate. All caregivers and children over 15 years of age signed an informed consent for participation. Inclusion criteria were bilateral CP and GMFCS level I–III. Twenty-four children were willing to participate. A pediatric orthopedic surgeon (first author, EPC) and a physiotherapist (ALZ) both well acquainted and experienced managing children with CP, were instructed how to use the 2D ML gait analysis traveled to the children’s local habilitation clinics to set up the equipment and perform the acquisitions.

### Follow-up program

Data from the most recent CPUP assessment, gender, age, height, weight and GMFCS level were obtained.

#### Gait classification and pattern description

The first (EPC) and last (JR) author, with 5 and 30 years of experience in pediatric orthopedic surgery respectively, together classified the children’s gait patterns according to the classification system of Rodda et al. [3]. The classification was made by using the acquired data from the 2D ML assessments, where the video images define the body segments and generate the graphs. The graphs from the hip, knee and ankle kinematics together with the foot orientation relative to the room (heel up or down) were analyzed at one session. A general observation of the gait from the video was the first step, then the graphs were analyzed separately, after these steps a final decision about the gait and the possible classification by Rodda et al. was made. Those that were not possible to classify according to Rodda, were still analyzed and described.

### Two-dimensional markerless gait analysis (2D ML)

**Fig. 1:**
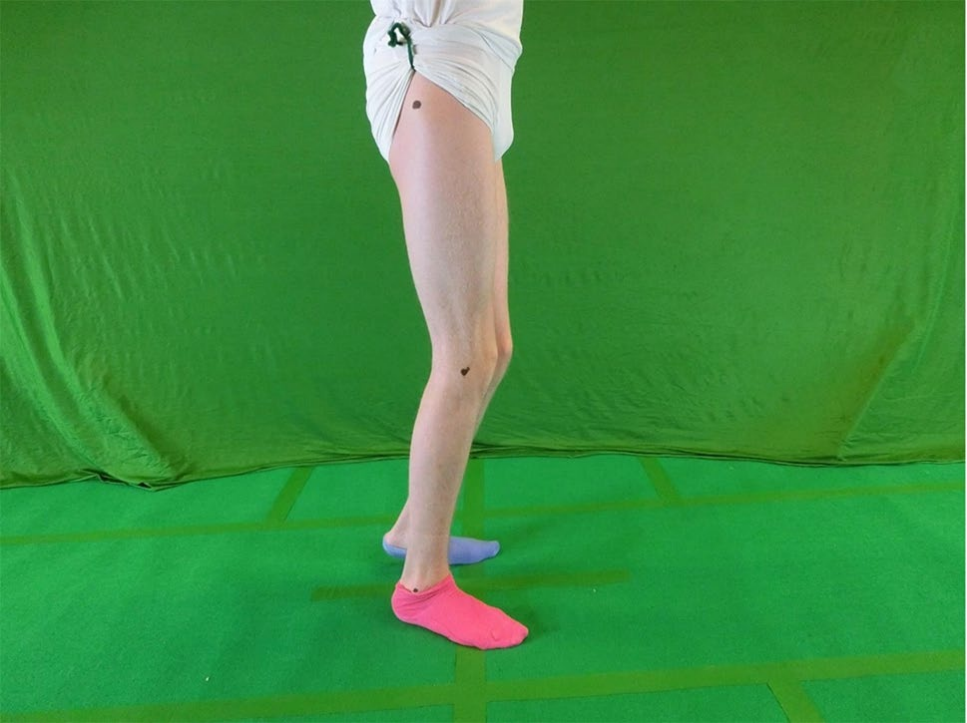
A static acquisition with skin marks on the greater trochanter, lateral epicondyle of the femur and on the lateral malleolus. Colored socks were used to define the foot segments

A previously developed 2D ML analysis system was used [31, 32, 35, 36]. The measurement system consisted of a red-green-blue-color camera combined with a depth-infrared sensor (RGB-D) (Kinect 2 for Xbox One, RGB images with resolution 1920 × 1080 pixels at 30 frames/second and depth image of 512 × 424 pixels at 30 frames/second). The camera was placed on one side of a walkway, with four light sources (to improve light conditions), and a green screen as background (to standardize the experimental scenario) on the other side. The setup was identical at each rehabilitation center. The child was asked to wear a t-shirt, underwear, and colored ankle socks, red for right foot and blue for left, to define the foot segment, see Fig. 1. For reference to bone anatomy, the locations of the greater trochanter, the lateral epicondyle of the femur and the lateral malleolus were marked on each leg with a black pen [31]. Static and dynamic acquisitions were performed. The participants walked at a self-selected speed on a 7-meter walkway regarded as enough space to walk at a steady state without the need for acceleration/deceleration. Depending on the walking capability and cooperation of the child, 2–6 gait cycles per side were recorded. Sagittal plane kinematics at the hip joint (approximated by the femur inclination relative to the horizontal line), knee, ankle joint (the tibia relative to the femur and foot relative to the tibia respectively) as well as the foot orientation in relation to the room and gait speed and step length were estimated. The calculation was performed by using MATLAB R2021a, The Mathworks, Natick, MA, USA on a laptop with Microsoft ^TM^ Windows 10 and Intel ® Core ^TM^ i7-6700HQ CPU @ 2.59 GHz.

The 2D ML video-based analysis has previously been validated against marker-based three-dimensional gait analysis, in both typically developed children and children with CP [31, 32, 37], showing average root mean square difference for hip, knee and ankle sagittal kinematics of 3.5, 3.2 and 4.5 degrees, respectively. In the present study, a comparison of gait speed and step length (computed as the distance between the first initial contact (IC) of the foreground and the subsequent IC of the background foot) parameters, as obtained from the automatic and manual procedures was performed by comparing the spatio-temporal parameters to values obtained through visual inspection of the RGB acquired frames. Specifically, initial foot contacts were identified both automatically (the heel and toe were automatically tracked by applying a color filter segmentation technique, IC was selected as the first instant of time between heel and toe characterized by zero-velocity) and manually via visual inspection of the RGB frames and were used to calculate spatio-temporal parameters, to explore the potential differences between the two methods.

## Statistics

The number of trials for each side and participant, along with the means with standard deviation and ranges for all trials for gait speed, step length and all 2-dimensional markerless analysis variables (hip flexion: femur inclination relative to the horizontal plane, knee flexion, ankle dorsiflexion and foot orientation in the room for the left and right side in each participant) were calculated using SPSS, version 22 (IBM Corp., Armonk, NY, USA).

## Results

### Demographics

Of the 24 participants, 3 were excluded since the images were not clear enough for correct identification of the feet (due to insufficient automatic exposure time) and 1 because a walking aid obscured a lower extremity. The remaining 20 children, 11 females and 9 males, mean age 10.4 (6.8–16.1) years, were included. Their mean height was 136 (119–159) centimeters and mean weight was 33 (19–63) kilograms. Eight were classified as GMFCS level I, eight as level II and the remaining four as level III. All children had bilateral CP.

### Two-dimensional markerless gait analysis (2D ML)

The four children with GMFCS level III needed support during data acquisition (crutches, cane, walker or holding hands).

The number of trials, gait speed, step length, hip, knee, ankle sagittal plane kinematics and foot orientation are presented in Table 1.

**Table 1:** The number of trials for each side and participant is given, along with the means of all trials for gait speed, step length and for all 2-dimensional markerless analysis variables (hip flexion: femur inclination relative to the horizontal plane, knee flexion, ankle dorsiflexion and foot orientation in the room for the left and right side in each participant). The mean, standard deviation (SD) and range for the total study cohort is given on the bottom rows. A minus (-) indicates a deficit from neutral. Four children used support: participant 7 used a cane and held another person’s hand, participants 14 and 17 each held another person’s hand and participant 19 used crutches for two trials and a walker for one

Participant	Side	Trials	Temporal spatial		Hip flexion		Knee flexion					Ankle dorsiflexion		Foot orientation
			*Gait speed* *m/s*	*Step* *length* *m*	*Max ext*	ROM*	*Initial* *contact*	*Loading response*	*Max* *in* *stance*	*Max* *in* *swing*	*ROM*	*Max* *in stance*	*Max* *in* *swing*	*Average* *10–30%* *of gait* *cycle*
1	L	5	0.86	0.46	-15	48	25	31	11	66	55	4	1	-0.8
	R		0.85	0.48	-14	50	31	33	15	70	55	5	-11	-2.5
2	L	4	0.85	0.43	-8	49	35	45	22	69	48	15	7	-5.4
	R		0.81	0.49	-19	56	33	41	11	62	51	16	5	3.6
3	L	2	0.86	0.40	-3	31	31	33	26	54	29	5	-8	5.6
	R		0.74	0.38	2	34	33	37	27	71	46	5	-15	3.5
4	L	5	0.91	0.42	-4	42	27	36	23	64	42	12	3	-5.2
	R		0.87	0.44	-5	42	43	46	33	65	32	17	2	-3.4
5	L	5	0.88	0.49	-10	47	31	31	17	69	53	7	-6	2.9
	R		0.81	0.49	-11	50	26	36	11	77	66	19	13	-2.8
6	L	4	0.72	0.25	5	37	50	63	53	69	24	14	-5	7.8
	R		0.70	0.48	14	30	57	67	62	74	22	-1	-9	21.1
7	L	3	0.13	0.12	8	21	38	38	30	45	14	-2	-7	6.9
	R		0.12	0.13	6	21	40	40	25	41	16	-4	-7	1.9
8	L	5	0.83	0.41	-10	42	17	31	16	68	58	12	-9	-6.1
	R		0.89	0.41	-13	40	12	27	11	68	62	12	9	-5.3
9	L	4	0.48	0.31	2	42	36	38	21	64	43	14	6	-3.9
	R		0.52	0.30	-6	37	30	31	16	74	58	11	5	-2.7
10	L	3	0.91	0.44	-4	39	39	41	29	66	37	16	3	0.8
	R		0.90	0.41	-10	45	32	36	14	76	62	16	2	0.5
11	L	3	0.64	0.32	-18	56	21	28	-4	66	70	17	1	-4.3
	R		0.61	0.39	-12	51	32	32	9	78	68	13	-5	0.8
12	L	3	0.87	0.45	-15	48	13	32	20	67	54	14	10	-5.9
	R		0.9	0.42	-18	49	16	31	14	62	51	7	3	-4.8
13	L	5	0.65	0.36	-11	46	34	37	14	51	39	7	-11	7.8
	R		0.58	0.34	0	53	48	52	28	80	52	0	-19	11.7
14	L	2	0.27	0.30	-14	52	58	58	21	71	50	20	-3	3.5
	R		0.30	0.29	1	36	34	35	10	65	57	11	-19	3.1
15	L	6	0.73	0.37	-4	49	23	33	25	72	59	14	4	-1.4
	R		0.81	0.36	-13	49	21	33	22	78	68	15	2	-1.7
16	L	4	0.60	0.36	-14	61	37	42	6	80	74	10	1	1.8
	R		0.62	0.35	-14	58	40	43	2	77	77	9	-5	1.7
17	L	2	0.52	0.31	-8	59	26	26	1	76	75	-19	-15	32.2
	R		0.27	0.24	-13	69	30	30	2	80	77	-10	-8	20.6
18	L	4	0.82	0.39	-25	64	23	42	5	76	71	11	-7	2.3
	R		0.86	0.38	-17	55	35	47	14	76	62	13	-7	-0.4
19	L	3			-13	46	10	10	-8	66	75	9	-13	
	R				-9	44	6	6	-9	68	77	13	3	
20	L	4	0.73	0.38	-15	56	31	43	10	70	59	12	2	-0.4
	R		0.74	0.40	-21	55	29	44	12	68	55	14	-3	-0.9
**Mean**			0.69	0.37	-9	46	31	37	17	68	54	9	-3	2.2
**SD**			0.22	0.09	8	11	12	11	14	9	17	8	8	8.0
**Range**			0.12–0.91	0.12–0.49	(-25)–14	21–69	6–58	6–67	(-9)–62	41–80	14–77	(-19)–20	(-19)–13	(-6.1)–32.2

*ROM-range of motion

#### Feasibility

On average, setup took 15 min, acquisition 10 min and data processing 50 min per child (6 walking trials with about 30 recorded frames each), which could be influenced by the child’s ability to cooperate and walk, which also determined the number of trials recorded: between 2 and 6 for each side. The equipment was easily movable by car to the five rehabilitation clinics in Sweden included, making the evaluation accessible for everyone. In addition, no need for special training to perform the acquisition was necessary. The total cost for the equipment including the camera was 800 $.

#### Gait classification and pattern description

**Fig. 2:**
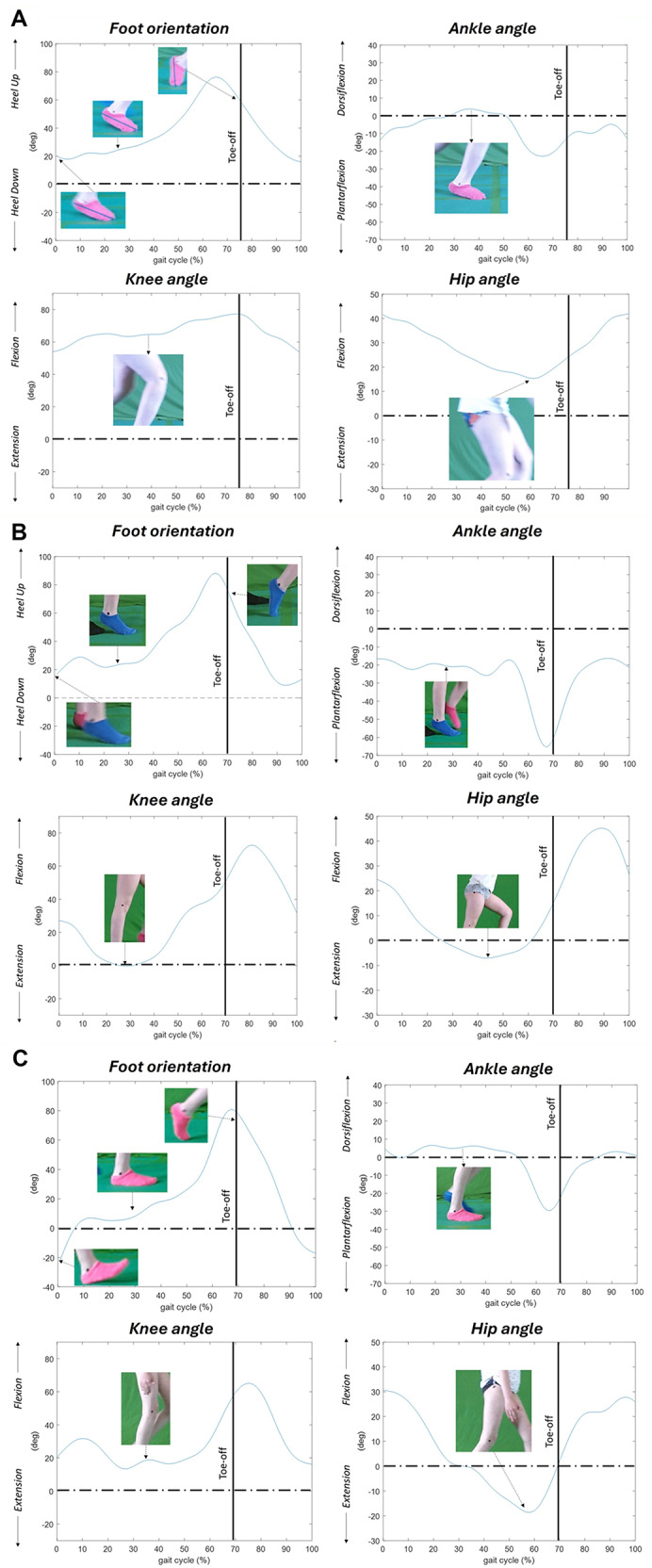
One gait cycle (x-axis 0-100%, with stance- and swing phase, divided by toe-off (vertical line). Foot orientation relative to the room with degrees of heel up/down (y-axis). Ankle kinematics with dorsiflexion/plantar flexion, and knee- and hip kinematics with flexion/extension. **A** Apparent equinus pattern in a 12-year-old boy (participant nr 6). There is an absent first rocker and during the time of the second rocker, the foot is not plantigrade; the child is toe-walking (in this case, with signs of a midfoot break). The ankle is in dorsiflexion in midstance and there is excessive knee- and hip flexion in stance. **B** True equinus pattern in a 7-year-old girl (participant nr 17). Toe-walking with ankle plantarflexion throughout the gait cycle, with increased plantarflexion at the end of the stance phase. The relative dorsiflexion motion into swing phase exhibits the capacity to perform dorsiflexion. Knee- and hip extension in stance. **C** Near normal gait pattern. 10-year-old girl (participant nr 12). Foot orientation (foot segment) relative to the room. The graph reveals an adequate first rocker 0–10%, second rocker 10–40% and third rocker 40% to toe-off

According to the Rodda classification, we could clearly distinguish two participants, one with apparent equinus and one with true equinus. Relevant data are shown in Fig. 2A and B where both graphs of the foot orientation and the ankle, knee and hip kinematics, as well as the imaging from the video is presented, for illustration.

Figure 2A (participant nr 6), 12-year-old boy, apparent equinus with toe-walking, without ankle plantar flexion and increased knee and hip flexion. Figure 2B (participant nr 17) a 7-year-old girl, illustrates true equinus pattern with toe-walking, ankle plantar flexion with knee and hip extension. Figure 2C (participant nr 12) a 10-year-old girl with near normal gait pattern is displayed for comparison.

In this cohort, the remaining 18 children exhibited limited deviations that were not possible to classify according to Rodda et al. Mild deviations were found in the kinematics from the hip, knee, ankle and foot orientation and the following are examples to illustrate the variability of the cohort.

**Fig. 3:**
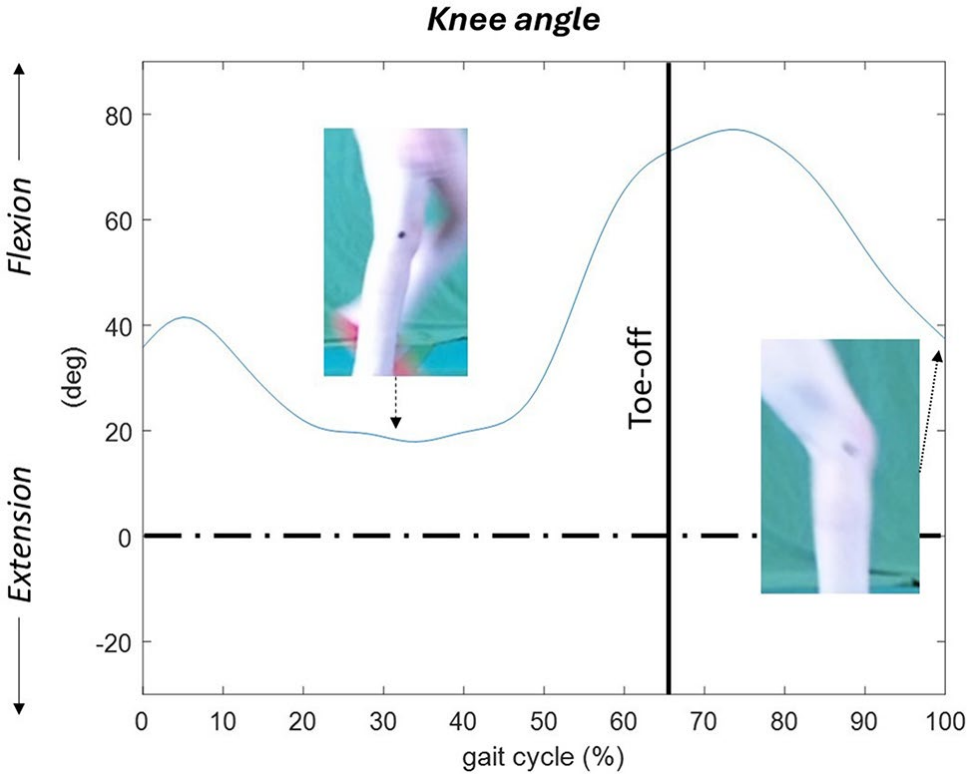
Participant nr 16, 7-year-old boy. Increased knee flexion at initial contact (0% of gait cycle) and decreased knee extension at terminal swing (90–100%), which could be signs of a short and/or spastic hamstring muscles. The kinematic pattern seems otherwise adequate, with around 60 degrees of range of motion

A 7-year-old boy, participant nr 16, exhibits increased knee flexion at initial contact, relative extension during stance that together with increased knee flexion at terminal swing, could possibly be a sign of short and/or spastic hamstring muscle Fig. 3.

**Fig. 4:**
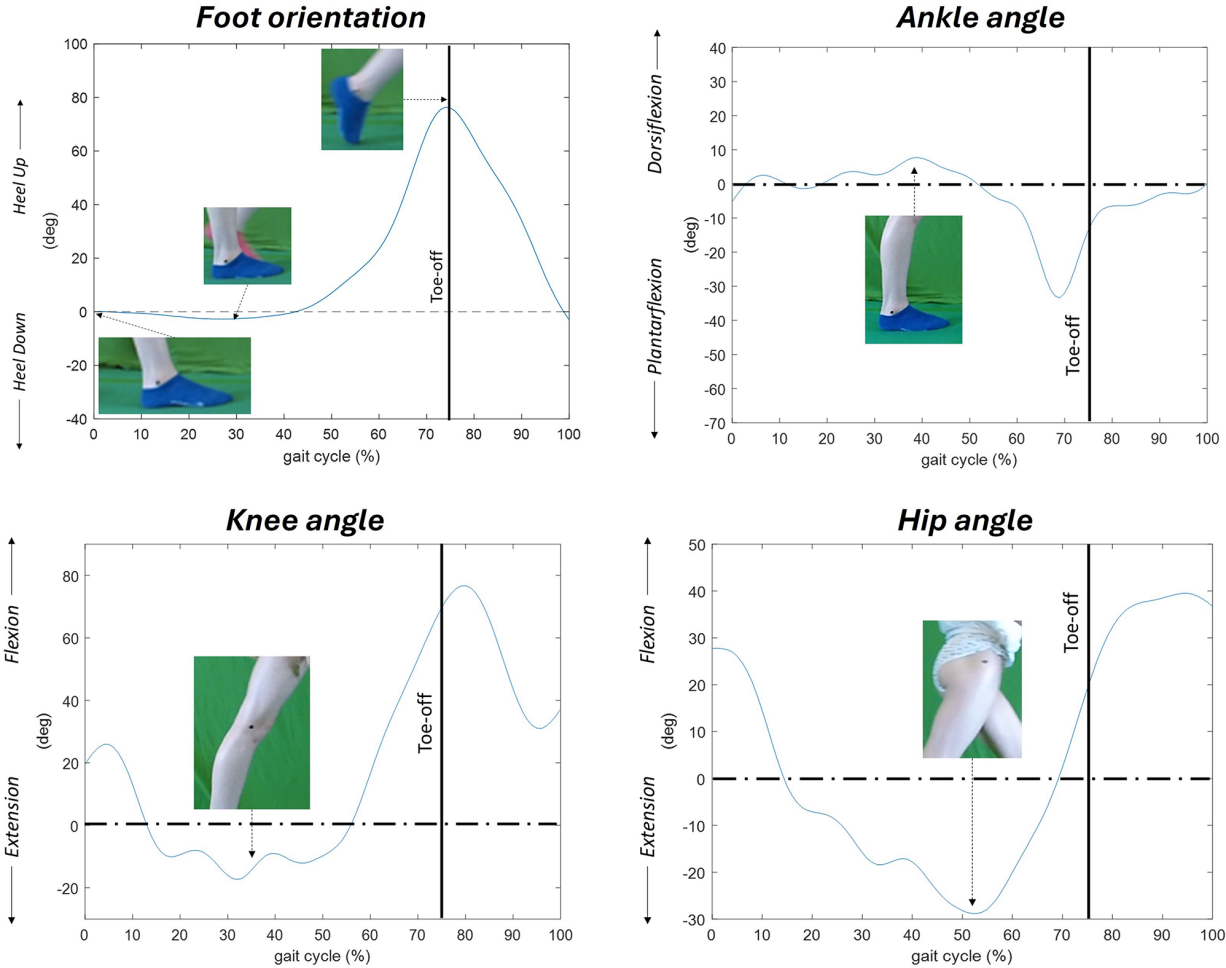
Participant nr 11, 10-year-old girl, with no first rocker, foot flat in stance. The ankle is in plantar flexion at initial contact, with limited dorsiflexion in stance. Excessive knee extension, as a sign of a strong “plantar flexion knee extension couple” (Rodda 2001, page 103). This means the gastrocnemius muscle/tendon complex, passing both the knee and ankle joint, strongly pushes the plantigrade foot towards the floor without the heel rising. Instead, the gastrocnemius muscle/tendon complex has an effect over the knee, that is driven into extension. In this case into hyperextension

Figure 4 reveals a 10-year-old girl with knee hyperextension in stance although no true equinus (plantar flexion) at the ankle. The increased knee extension is most likely a result of a tight or spastic gastrocnemius muscle/tendon complex, which results in a strong plantarflexion knee extension couple, not uncommon in the young child with bilateral CP.

#### Gait speed and step length

**Fig. 5:**
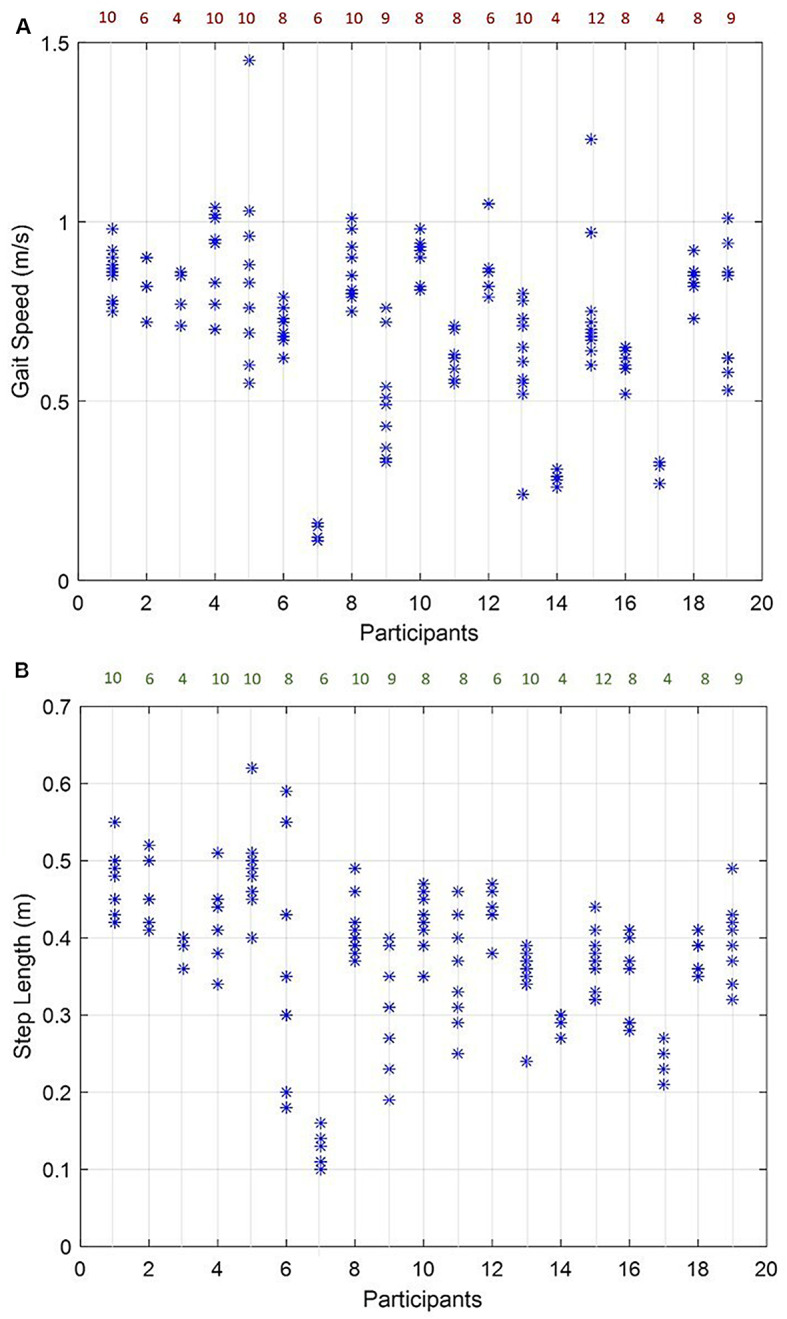
**A** Gait speed distribution in meters/second (y-axis) for the 20 participants for all of their trials (x-axis). The numbers at the top are the total number of trials for each participant (left and right). **B** Step length distribution in meters (y-axis) for the 20 participants for all of their (x-axis). The numbers at the top are the total number of trials for each participant (left and right)

Gait speed and step length for all 20 participants are presented in Table 1 and illustrated in Fig. 5A and B, respectively. Mean and standard deviation of absolute error was 0.002 m/s (0.006) for gait speed and 0.015 m (0.006) for step length.

## Discussion

In this study, we used the 2D ML video-based analysis system, at several local habilitation clinics in Sweden, to explore its feasibility and possible usefulness in evaluation of gait in children with CP. We identified and quantitatively assessed several minor and major deviations in hip, knee, ankle kinematics and foot orientation, as well as in gait speed and step length.

### Feasibility; setup, acquisition and processing

The measurement system, which included a single camera, light sources, walkway, and background, was easy to setup. The equipment can be moved to any location and the room required for 2D ML is smaller than for most 3D GA systems.

Acquisition always depends on the child’s cooperation and walking ability, but the 2D ML system saves time compared with a 3D GA. The 2D ML also has the advantage of no markers being attached to the body, which can be uncomfortable for the child and are at risk of falling off during acquisition.

The processing is automated, requiring limited manual involvement, and thus saving time. Another advantage is that 2D ML does not require specific training or technical knowledge. This, together with low cost of equipment and that the system is portable, makes it suitable for screening and follow-up purposes.

### Gait classification and pattern description

The classification system of Rodda et al. is based on kinematics in the sagittal plane, however, acknowledge the importance of coronal and transverse plane deviations in children with CP and a 3D GA is required for a full gait evaluation of all planes [3]. Although the coronal and transverse plane are important for a full gait evaluation, with the purpose of evaluating the sagittal plane and classify according to Rodda et al., the 2D ML system may be a good compromise between completeness and feasibility [3, 32].

Based on ankle kinematics and foot orientation, we could identify two of the gait patterns described by Rodda et al. and present in our cohort: true equinus (Fig. 2B) and apparent equinus (Fig. 2A). In the 7 years old girl with true equinus gait pattern (Fig. 2B), toe-walking with ankle plantarflexion throughout stance is prominent with hip and knee in full extension, which is what was used to differentiate true from apparent equinus in our cohort. This pattern is often caused by calf muscle spasticity and can be managed by spasticity reduction (Botulinum toxin injections), hamstring lengthening surgery and/or ankle-foot orthosis. True equinus is common in the young child, but rarely remains throughout childhood [3, 4]. The 12-year-old boy with the apparent equinus gait pattern (Participant nr 6, Fig. 2A), toe-walking is also present, however, the ankle has a normal ROM and it is increased knee and hip flexion during the stance phase that causes toe-walking (apparent equinus). It is crucial to distinguish apparent equinus from true equinus since the former seldom requires surgical treatment with calf muscle lengthening, which is commonly performed in true equinus [3, 4]. In case the calf muscle is lengthened surgically, especially if the achilles tendon is lengthened, there is a high risk of future crouch gait, a complication notoriously difficult to treat. In addition to the gait analysis, careful assessment is needed through physical examination to distinguish between short gastrocnemius muscle only and if both the gastrocnemius and soleus muscles are short, to determine the level of calf muscle lengthening required. Hence, it is important to perform comprehensive assessments of children with CP, in this case combine the dynamic assessment with gait analysis with a more static physical examination, where in the later both muscle/tendon length and muscle tone is included.

It should be highlighted that the commonly developed foot deformity with equinus, forefoot abduction and pes planovalgus with midfoot break are especially problematic and difficult to assess reliably even using 3D GA or 2D ML systems. In fact, it can be difficult to identify if a child toe-walks or not and if the ankle is in plantarflexion or in dorsiflexion since this may be obscured by the foot deformity. Nevertheless, we found the foot orientation in the sagittal plane, described in this study, helpful.

Rodda et al. described a mild gait deviation pattern: sagittal kinematics within the 1 SD band of their laboratory normal range [9]. We also identified mild gait deviations in our cohort (Figs. 3 and 4).

Although the hip kinematics with the 2D ML system used in this study was based on the thigh in relation to a horizontal line, not in relation to the pelvis, as in the 3D GA system, the gait patterns described by Rodda and Graham could be determined almost fully. Furthermore, even with the offset between the 2D ML and 3D GA systems, it was possible to determine and identify deviating patterns with the 2D ML method, making comparisons over time possible. Additionally, it has been pointed out that performing classification of gait patterns aids prognosis, enabling awareness of expected later deviations of gait pattern [3, 38].

It may be useful to identify a strong plantar flexion knee extension couple, since if not treated with time, may result in a permanent knee hyperextension gait pattern, depending on an overly stretched posterior knee capsule, being difficult to treat with a poor prognosis for continued walking capacity.

### Flexed knee gait

The 2D ML could possibly aid in early identification of increasing flexed knee gait over time, since it quantifies the degree of flexion in stance, and may be used on a regular basis as a screening tool. Thus provide a possibility for early intervention, with specific further assessment and adequate treatment, such as of physiotherapy, orthosis and orthopeadic surgery.

### Gait speed and step length

Both subjective perceptions of gait and the ability to control gait speed have been reported in children at various GMFCS levels [39, 40]. In our cohort, the mean gait speed was 0.69 m/s (range 0.12–0.91), far from the average gait speed in typically developed children, where a 7-year-old walks at an average of 1.14 m/s [41].

Adequate step length is considered a prerequisite for acceptable gait speed, with associated stability in stance [23, 42]. Abel et al. reported shorter step length, 0.79 m (SD 0.19) in children with bilateral CP compared with typically developed children, 1.08 m (SD 0.14) [43]. Our cohort had even shorter step length, mean 0.37 m (SD 0.08, range 0.12–0.49).

### Follow-up programs and limited assessment of gait

In most follow-up programs for children with CP, extensive physical examinations are performed on a regular basis with a focus on preventing severe complications, such as hip dislocation, common at GMFCS levels IV and V. However, around 70% of children with CP are at GMFCS levels I, II or III and are ambulatory [12]. Nevertheless, no objective quantification of gait variables is currently included in the follow-up programs, to screen and follow the development of gait over time. Static physical examination alone has been shown to be insufficient to assess gait [44, 45].

Haumont et al. pointed out in a study from a pediatric specialty center that a treatment program with careful orthopedic follow-up based on medical history, physical examination and gait analysis leads to improved gait function throughout childhood [37].

## Limitations

Limitations include the small number of participants in the study and that we did not collect data on typically developed children. Additionally, 3 out of 24 subjects did not have sufficiently clear images for accurate foot identification. In the 2D ML we did not evaluate the transverse or coronal plane, both of which are important in the evaluation of gait deviation in children with CP.

Another clear limitation is that a 3D GA was not performed and thus a comparison with the classification by Rodda et al. [3] between the two systems was not possible. As well we did not follow our study cohort over time, although this study did establish 2D ML gait analysis as a possible dynamic complement in the evaluation of gait. Nevertheless, we think these children can serve as examples, illustrating various variables and deviations possible to obtain with the 2D ML technique and the practicalities surrounding the assessments and processing of data.

## Conclusion

The 2D ML is feasible, inexpensive, provides objective measurements, and does not require advanced knowledge for performing assessments. It would be both relevant and reasonable to include an objective and quantitative analysis of gait in follow-up programs for children with CP, to detect deviations and progression. Consecutive measurements are needed to evaluate the suitability of the 2 D ML system for follow up.

## Data Availability

The datasets used and/or analyzed during the current study are available from the corresponding author on reasonable request.

## Abbreviations

CP: Cerebral palsy
2D ML: 2 dimensional markerless
3D GA: 3 dimensional gait analysis
GMFCS: Gross motor function classification system
CPUP: The cerebral palsy follow-up program
